# How PNIPAM Microgel Architecture Controls Pickering Foam Formation

**DOI:** 10.1002/smll.202513819

**Published:** 2026-02-02

**Authors:** Antoine Brézault, Anne R. Rousseau, Véronique Schmitt, Valérie Ravaine, Patrick Perrin, Nicolas Sanson, Cécile Monteux

**Affiliations:** ^1^ Centre de Recherche Paul Pascal, Université de Bordeaux CNRS UMR 5031 Pessac France; ^2^ Bordeaux INP, ISM, Université De Bordeaux CNRS UMR 5255 Talence France; ^3^ Soft Matter Sciences and Engineering, ESPCI, PSL University Sorbonne Université, CNRS UMR 7615 Paris France; ^4^ Institut De Recherche de Chimie Paris (IRCP), Chimie ParisTech PSL Research University, CNRS Paris France; ^5^ Agence de l'Environnement et de la Maîtrise de l'Energie (ADEME) Angers France

**Keywords:** adsorption kinetics, interfaces, microgel, pickering foam, structure‐activity relationships

## Abstract

The generation of foams using particle‐like objects has gained momentum in the past years as such Pickering foams are relevant in many industrial applications. In this context, we investigate the foamability of microgels, which are soft polymeric particles which architecture can be finely tailored during synthesis. Herein, foams were produced by continuously bubbling air into dispersions of poly(*N*‐isopropylacrylamide) (PNIPAM) microgels. The foaming ability of the microgel dispersions has been assessed by combining observations at both the macroscopic and the local scales. Increased microgel concentration and more pronounced core‐shell structure lead to a higher foamability, smaller bubbles, and wetter foams. In contrast, microgel size variation has a small effect on the foam properties. These results are correlated to the adsorption kinetics assessed by pendant drop tensiometry. Indeed, faster adsorption kinetics are expected to promote larger surface coverage during bubble formation, which increases its stability against coalescence and the subsequent ability of the microgel dispersion to generate large volumes of foam. Such a hypothesis is confirmed by salt addition, which accelerates microgel adsorption and thus enhances foamability.

## Graphical abstract

Foams were generated by bubbling air into dispersions of PNIPAM microgels. We show that microgels with a more pronounced core‐shell architecture exhibit higher foamability: larger volume of foams, with smaller bubbles and a higher liquid fraction are produced. These observations are correlated with faster adsorption of microgels at the interface.

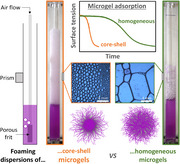

## 1 Introduction

Aqueous foams are concentrated dispersions of gas bubbles in water, creating large interface areas that need to be stabilized [1, 2, 3]. Industrial applications often require high foamability and long foam stability, which are difficult to conciliate [4, 5, 6]. Thus low‐molecular weight surfactants grant good foamability thanks to their fast adsorption kinetics yet lead to fast foam aging due to reversible adsorption. In contrast, hard particles have high adsorption barriers which impede foam formation but ensure long lifetime once adsorbed [7, 8, 9]. Such particles are commonly found in industrial applications, such as proteins aggregates in the food industry [10, 11, 12] or hard mineral particles in froth flotation [13, 14, 15].

In that context, PNIPAM microgels, which are soft particles composed of a macromolecular crosslinked network that swells in water [16], could be useful to generate foams. Indeed, these microgels spontaneously adsorb to the air/water interface thanks to the amphiphilic character of PNIPAM chains, as proven by pendant drop tensiometry [17, 18], so that foams can be simply generated by hand‐shaking [19, 20]. Adsorption is accelerated by increasing the microgel concentration, by reducing the crosslinker content, and by reducing the electrostatic repulsion between microgels [18]. During adsorption, thanks to their softness, microgels deform at the interface and their shells entangle, which increases surface elasticity [21, 22, 23, 24]. Besides, microgel adsorption is irreversible due to the “particle” like behavior which endows them with an anchoring energy much larger than the thermal energy [18, 25], providing long term stability for microgel‐stabilized emulsions and foams [26, 27, 28, 29].

From a fundamental point of view, using soft particles to stabilize foams is also of great interest since their size, shape, softness, structure, and hydrophilic/hydrophobic balance can be precisely tuned during their synthesis [26]. Thus, it has been established for oil‐in‐water emulsions that emulsion properties are linked to an intricate interplay between the microgel structure (that is to say the crosslinker spatial distribution), their adsorption kinetics, the emulsification process, and the resulting interfacial conformation [18, 27, 30, 31, 32]. Similarly, it is expected that these parameters should be of crucial importance to understand the microgels role in foam formation and stabilization.

The microgels behavior at air‐water interfaces has been investigated by some authors at the scale of a single film [33], while others have exploited the thermoresponsiveness of PNIPAM microgels [16, 34, 35] to trigger the foam destabilization with increasing temperature [36, 37, 38]. Recently, Kühnhammer et al. systematically investigated the impact of PNIPAM microgels characteristics on foam properties [20]. In particular, they have varied the microgels crosslinking ratio to assess the impact of the microgels softness on the foam stability as it was shown to strongly impact the stability of emulsions, where higher crosslinker content leads to lower stability [27, 39]. Conversely, foam stability seems to increase at high microgel crosslinking density, perhaps due to aggregation phenomena within the liquid films, preventing drainage [20]. This hypothesis was supported by neutron scattering experiments as well as thin film pressure balance measurements, which both proved high thickness heterogeneities within films [20]. This example also proves that, despite some similarities with emulsions, foams stabilized by microgels have original properties probably due to these two main differences: the process of formation and enhanced drainage. Besides, varying the crosslinking ratio modifies both the microgels size and structure, which respective behavior in foams have not been reported in the literature to our best knowledge. Furthermore, while considerable effort has been devoted to improving foam stability, only a few studies have focused on foamability, even though the initial structure of the foam determines how it ages [40, 41, 42].

In the present article, PNIPAM‐based microgels of independently tuned size and crosslinker distribution were synthesized to systematically study the impact of the microgel architecture on foam properties during foam generation. In this study, we use a supramolecular crosslinker exhibiting a characteristic visible absorption that results in a pink coloration in solution. Its high electron density enables quantitative determination by visible spectroscopy and direct visualization of its spatial distribution by transmission electron microscopy. In the first part, we investigate the structure of a foam stabilized by microgels during its generation and evolution over time, at local and global scales. We then investigate how to improve foamability by playing on microgels concentrations and on a large range of microgel structures. We demonstrate that foamability parameters are mainly governed by microgels adsorption kinetics, and confirm it by tuning the ionic strength of the foaming solution.

## 2 Results and Discussion

### 2.1 Independent Variation of Microgels Size and Structure

**SCHEME 1:**
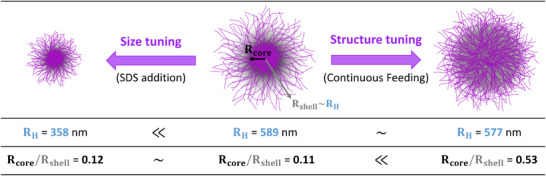
Controlled microgel architecture: independent variation of microgel size and structure. Note that the used supramolecular crosslinker endows microgels with a pink color, explaining the color of the liquid dispersions presented in the following.

The foamability and foam stability are investigated with two series of PNIPAM‐based microgels, referred to as size and structure sets, where the size and the structure of microgels are independently modulated keeping the crosslinker content close to 1 mol% *vs* NIPAM [43, 44] (Scheme 1, see Supporting Information for details on microgels synthesis—Figure S0). As expected from the literature [45, 46], microgel radii in the collapsed state R_H_(70°C) and in the swollen one R_H_(20°C) decrease for an increasing sodium dodecyl sulfate content. Thus, in the size set, microgel radii in the swollen state are tuned from R_H_(20°C) = 708 to 358 nm while the internal structure, that is, the core to (core + shell) ratio, evaluated either by Transmission Electron Microscopy (TEM) or by Atomic Force microscopy (AFM) stays constant with small values between 0.08 and 0.12, indicating a constant “ultra” core‐shell structure (Figure S1, Table S1). In the following, microgels from the size set are referred to as µG‐R‐*xx* where *xx* stands for the hydrodynamic radius measured at 20°C. On the contrary, in the structure set, the crosslinker distribution in microgels is tuned by continuously feeding the crosslinker [47, 48, 49]. In that case, one can observe a variation of the core to (core + shell) ratios from 0.11 to 0.53, while the R_H_(20°C) does not significantly vary (Figure S1, Table S1). In the following, microgels from the structure set are referred to as µG‐CS‐*yy* where *yy* stands for the core to (core + shell) size ratio assessed by TEM.

### 2.2 Foamability of a Core‐Shell Microgel Dispersion and Resulting Foam Stability

**FIGURE 1:**
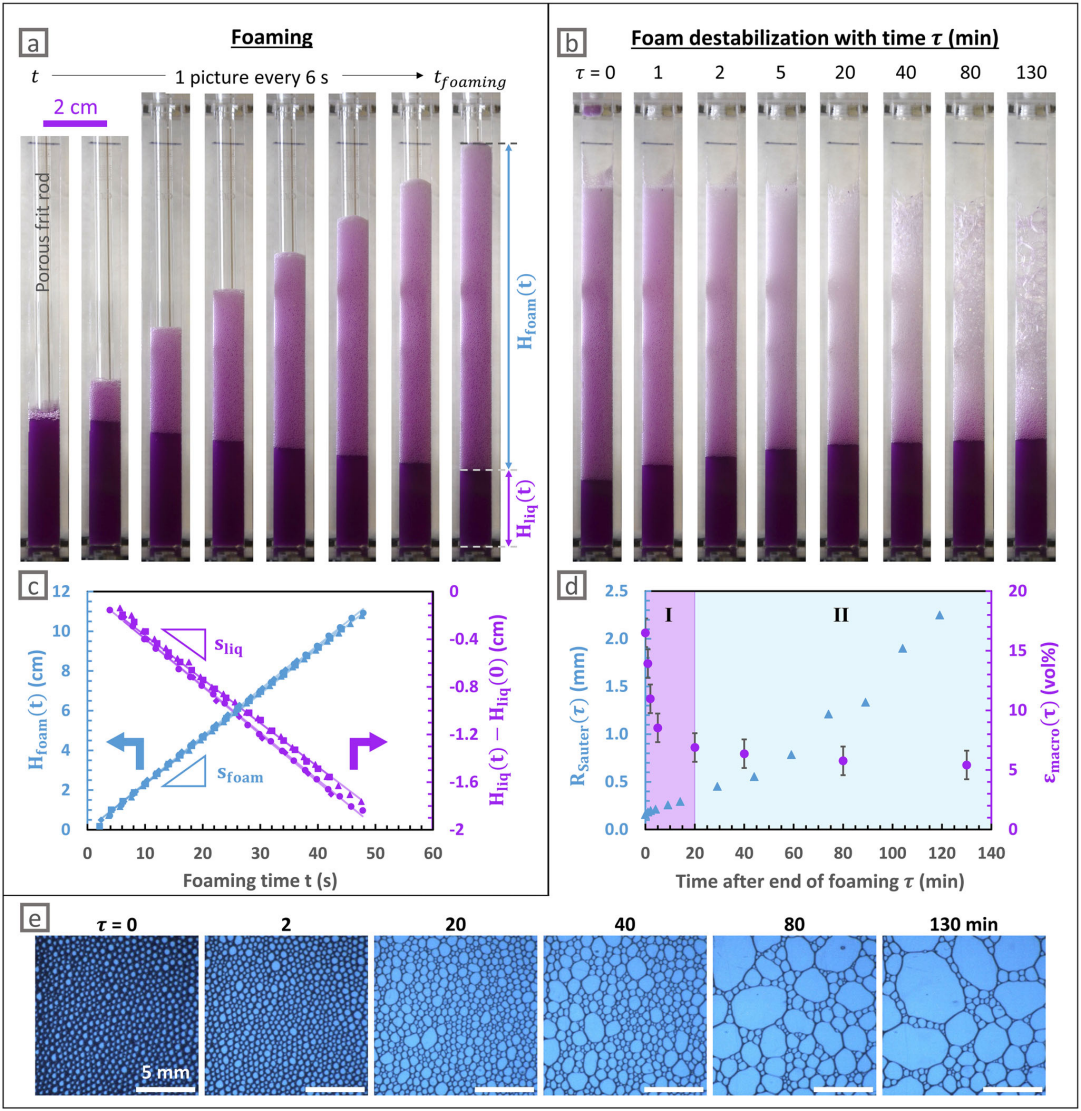
Data acquired during the generation and destabilization of foams formed by bubbling air at 20 mL min^−1^ through a porous frit in a dispersion of µG‐CS‐0.1 microgels at 3 mg mL^−1^ at ambient temperature. Macroscopic pictures taken during (a) foam generation and (b) after foam formation at times τ = *t* − *t_foaming_*. (c) Time evolution of foam height (H_foam_(*t*)) and liquid height below the foam compared to its initial height (H_liq_(*t*) − H_liq_(0)) during foam generation. Foam and liquid height evolution were assessed in four experiments, and modeled with a linear regression, giving *s*_foam_ = 0.232 ± 0.005 cm.s^−1^ and s_liq_ = −0.038 ± 0.003 cm.s^−1^. (d) Average Sauter radius R_Sauter_(τ) of the bubbles and macroscopic average liquid fraction $\varepsilon_{\mathrm{macro}}\left(\tau \right)=\frac{H_{\mathrm{liq}}\left(0\right)-H_{\mathrm{liq}}\left(\tau \right)}{H_{\mathrm{foam}}\left(\tau \right)}$ after foam formation at times τ = *t* − *t_foaming_*. (e) Local images of the foam taken after foam formation at times τ = *t* − *t_foaming_* through the right‐angle prism placed at a 6.5 cm height. Scale bar is always 5 mm.

To investigate the foamability of microgel dispersions, foams were generated by bubbling air through a porous frit at a constant flow rate of 20 mL min^−1^ in a 3 mg mL^−1^ µG‐CS‐0.1 microgel dispersion. Macroscopic images of the foams were recorded to monitor the evolution of both the foam and liquid heights over time during foam generation (*t* ≤ *t_foaming_*, porous frit rod inside the column, Figure 1a) and during subsequent foam destabilization (τ ∼ *t* − *t_foaming_*, fixed to 0 after the end of foaming and the subsequent removal of the porous frit, Figure 1b). First, Figure 1a,c show that the foam height H_foam_ increases over time while the liquid height H_liq_ decreases with time. A foam height of 10 cm, corresponding to 20 cm^3^ of foam, was reached in *t_foaming_* = 45 s, clearly demonstrating the ability of the µG‐CS‐0.1 to efficiently entrap air and generate large amount of foams, as expected from the spontaneous adsorption of PNIPAM microgels to air/water interfaces [17, 20]. Note that interfacial tension analyses revealed the absence of any residual impurity that would be able to adsorb faster than the microgels themselves (Figure S2). Besides, we highlight that this foaming process allows reaching 7 cm of foam height—corresponding to 14 cm^3^ of foam—in 30 s using 8 mL of microgels dispersion, whereas, using the Bartsch method [50], Kühnhammer et al. reported a maximum foam height of 1.5 cm [20], corresponding to 9 cm^3^ of foam, when shaking the same volume of microgels dispersion during 30 s. This change may be attributed both to the modification of the processing conditions and to the use of PNIPAM microgels conventionally crosslinked with methylene bisacrylamide (BIS), as discussed below.

Figure 1c presents the superposition of four independent foaming experiments (shown as four different symbols), demonstrating that the time evolution of both the foam and liquid heights is highly reproducible and confirming the robustness of the foaming process. The foam and liquid heights evolve linearly as a function of the foaming time *t*. Modeled slopes s_foam_ = 0.232 ± 0.005 cm.s^−1^ and s_liq_ = −0.038 ± 0.003 cm.s^−1^ allow the calculation of the flow rate at which foam is produced (Q_foam_ = s_foam_S = 0.39 ± 0.01 cm^3^.s^−1^) as well as the flow rate at which liquid is withdrawn (Q_liq_ = s_liq_S = −0.06 ± 0.01 cm^3^.s^−1^), where S = 1.67 ± 0.04 cm^2^ is the free section of the column, that is, the one obtained by withdrawing the section occupied by the porous frit cylinder. The ratio of these two slopes gives the average liquid fraction of the foam ε_macro_ =  |s_liq_|/s_foam_ =  16 ± 1 vol% during foaming, that is, the total volume of liquid inside the foam divided by the total volume of foam. Such a high liquid fraction is expected just after the foam generation when bubbling into a stationary liquid, as bubbles are generated directly in the liquid and carry liquid while rising [51].

Once the foam reached the desired height (10 cm), air injection was stopped, and the porous frit was removed. Note that the apparent change in foam volume between the picture taken at the end of foaming (*t* = *t_foaming_*, Figure 1a) and the picture taken at the beginning of the destabilization study (τ = 0, Figure 1b) is due to the removal of the porous frit. The subsequent evolutions of liquid and foam heights were monitored over time with macroscopic images (Figure 1b). Remarkably, the half‐life of the foam, defined as the time at which the foam volume had been divided by two, is still not reached after 130 min, confirming the ability of the µG‐CS‐0.1 to stabilize foams.

It can also be seen on Figure 1b that the height of the liquid below the foam increases with τ, indicating that the continuous phase drains because of gravity. An example of the time evolution of the macroscopic average liquid fraction of the foam after the cessation of foaming process, $\varepsilon_{\mathrm{macro}}\left(\tau \right)=\frac{H_{\mathrm{liq}}\left(0\right)-H_{\mathrm{liq}}\left(\tau \right)}{H_{\mathrm{foam}}\left(\tau \right)}$, is plotted in Figure 1d. In addition, we also monitored the average Sauter radius of the bubbles R_Sauter_(τ), plotted as a function of τ in Figure 1d, and measured from local pictures of the foam presented in Figure 1e as detailed in the Experimental Section in Supporting Information. Two regimes are observed (I and II, Figure 1d). First, the macroscopic average liquid fraction decreases rapidly with time, from 16 to less than 7 vol% in about 20 min. In the meantime, the average Sauter radius of the bubbles slightly increases from 0.2 to 0.3 mm, while the total volume of foam plus liquid stays approximately constant (Figure 1b). Thus, the foam aging is dominated by drainage in regime I at short times. At longer times, there is a quick increase in mean bubble size from 0.5 to 2.3 mm between 30 and 140 min, while the liquid fraction stays stable at around 6 vol%, which tends to prove that coalescence and coarsening become prevalent over drainage in regime II.

The analysis of the local images presented in Figure 1e also allowed us to record the local surface liquid fraction ε_loc_ (proportion of the images surface area corresponding to the liquid phase) plotted in Figure S3. It should be noted that ε_loc_ is shown as a surface liquid fraction and ε_macro_ is a volume liquid fraction so that these two measurements of the liquid fraction cannot be directly compared (see II.1 in Supporting Information for more explanations). Nevertheless, this local information not only illustrates but also confirms the liquid fraction decrease due to drainage.

In summary, these results demonstrate that stable foams can be efficiently produced by bubbling air into a microgel dispersion. This foaming process not only generates large volumes of foam that remain stable for several hours, but also enables the assessment of the foam volume over time. During foaming, the evolution of foam and liquid heights is macroscopically analyzed while the bubble size is locally characterized at the end of the foaming process. Using this approach, we can explore the effect of microgel concentration on the foaming behavior, shedding light on the specific role of the microgels in the foaming process.

### 2.3 Microgel Concentration Effect on Foamability and Foam Stability

**FIGURE 2:**
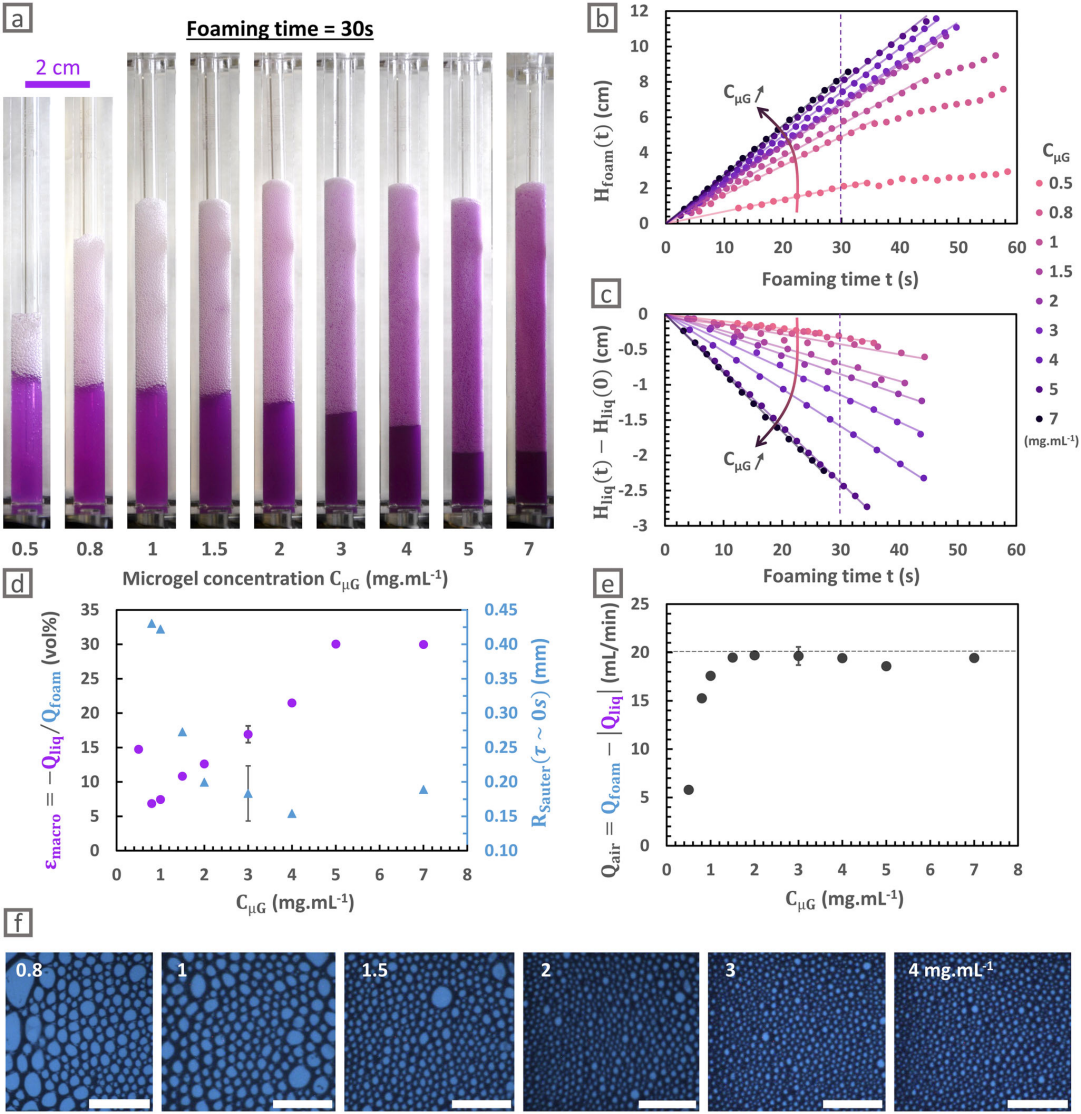
Effect of the microgels concentration C_µG_ of µG‐CS‐0.1 on foam generation. (a) Macroscopic pictures at t = 30 s during foam generation for different C_µG_ in mg mL^−1^. (b) Height of the foam during foaming (H_foam_(*t*)) for different C_µG_ in mg mL^−1^. (c) Difference between the liquid height at times *t* and *t* = 0 (H_liq_(*t*) − H_liq_(0)) as a function of the foaming time t for different C_µG_ in mg mL^−1^. (d) Average macroscopic liquid fraction ε_macro_, determined from the slopes in the linear regime of the foaming process, and the average bubble radius R_Sauter_, determined at the end of the foaming process on images presented in f, as a function of C_µG_ in mg mL^−1^. (e) Apparent flow rate of the air Q_air_, corresponding to the flow rate of air efficiently trapped in the foam in the linear regime as a function of C_µG_ in mg mL^−1^. The dashed line represents the injected air flow rate of 20 mg mL^−1^. (f) Local images of the foams taken at the end of the foaming process for different C_µG_ in mg mL^−1^. Scale bar is always 5 mm.

The previously presented bubbling method was used to foam dispersions of the same microgel (µG‐CS‐0.1), varying concentrations C_µG_ between 0.5 and 7 mg mL^−1^. Air was thus continuously injected through the porous frit until the foam reaches the top of the column or, if not possible, any other lower stationary height. Photographs were taken every 2 s during foaming. For a fixed bubbling time (*t* = 30 s), a larger volume of foam is created for larger C_µG_ (Figure 2a). From the photographs, we can also qualitatively see that the amount of excess liquid decreases when C_µG_ increases.

On a more quantitative level, the evolutions of foam and liquid heights during foaming were measured as a function of the foaming time t and reported in Figure 2b,c respectively. A linear regime is still observed at the beginning of the foaming, regardless of the dispersion concentration. The slopes s_foam_ and s_liq_ determined in this regime as defined in the previous part, are reported in Figure S4. Corresponding foam and liquid flow rates, Q_foam_ and |Q_liq_|, increase with C_µG_, which shows that air is more efficiently trapped and that more liquid is incorporated in the foam for higher microgel concentrations. We extracted the average macroscopic liquid fraction of the foams ε_macro_ =  |s_liq_| / s_foam_ which increases from 7 to 30 % when increasing C_µG_ from 0.8 to 7 mg mL^−1^ within this initial linear regime (Figure 2d).

Besides, the images of the foam surface at the column wall allowed the determination of the average bubble Sauter radius R_Sauter_ for different C_µG_ at the end of the foaming process (Figure 2d,f). R_Sauter_ decreases from 0.45 to 0.15 mm when increasing C_µG_ from 0.8 to 4 mg mL^−1^, and then seems to level off. It is worth noting that a similar behavior has been reported for protein‐stabilized foams, where a critical concentration exists above which the size of the bubbles no longer evolved under a fixed foaming process [52].

In summary, increasing the microgel concentration enables to decrease the bubble size, obtain wetter foams that is, with a higher liquid volume fraction, and produce larger foam volumes. In the following, we discuss these results point by point by considering (i) the hydrodynamics at play during the air ejection from the pores, which controls the initial bubble size, and (ii) the bubble coalescence and liquid drainage that may occur as the bubbles rise in the liquid column and accumulate at the top of the column. These phenomena are expected to depend on microgel concentration, since it affects both interfacial and hydrodynamic properties, as discussed below.

We first address why the bubble size decreases when the microgel concentration increases. Higher concentrations are known to induce faster adsorption kinetics [17, 18], as seen by the quicker decrease in surface tension measured by pendant drop tensiometry (Figure S5). Note that concentrations as large as the ones used for foaming dispersions cannot be directly probed, as pendant drop tensiometry cannot precisely detect adsorption faster than 1 min. Yet, we hypothesize that the trend should be the same at higher concentrations. Thus, faster adsorption of microgels at higher concentrations would decrease the surface tension more quickly and lead to an earlier detachment of the bubble [51], which would consequently be smaller (mechanism i). In addition, the accelerated microgel adsorption is also expected to increase the surface coverage of bubbles, providing enhanced protection against coalescence events and thus limiting the bubble growth (mechanism ii).

Second, we rationalize the increase in liquid fraction due to reduced drainage, when C_µG_ increases. We observed that bubbles tend to be smaller if microgel concentration is increased (Figure 2d), and smaller bubbles are known to reduce the drainage rate [1, 2]. Moreover, a higher microgel concentration is expected to increase surface coverage, which would decrease interfacial mobility [53, 54]. This reduced mobility would also reduce the drainage rate. Finally, for larger C_µG_, more interactions between the microgels trapped in the films and in the Plateau borders could be expected, which would locally increase the liquid viscosity [55] and again reduce drainage [56].

Third, we explain why foam volume increases when microgels concentration increases. For foams stabilised by microgels at concentrations higher than 1.5 mg mL^−1^, we see that there is no gas loss during foaming (Figure 2e). Indeed, the measured flow rate of air incorporated in the foam Q_air_ = Q_foam_ −  |Q_liq_| reaches the injected air flow rate of 20 mL min^−1^ for C_µG_ larger than 1.5 mg mL^−1^, proving that all the injected air is efficiently trapped in the foam. Therefore, the difference in foam volumes made with high C_µG_ results only from the difference in their liquid content. On the contrary, foams made from microgels dispersions less concentrated than 1.5 mg mL^−1^ show significant gas loss (Figure 2e), indicating that coalescence events happen at the top of the foam during foaming. As explained before, foams made with lower concentrations exhibit larger bubble size, lower liquid fraction, and possibly lower microgel surface coverage. All these factors are known to increase the probability of bubble coalescence. Indeed, the coalescence probability is expected to increase with the bubble area [57, 58, 59, 60] whilelower liquid fraction might induce thinner films, which are more prone to hole nucleation leading to their rupture [61]. Moreover, possibly lower surface coverage would not confer sufficient protection and might therefore favor coalescence events.

For longer foaming times and C_µG_  ≤ 1.5 mg mL^−1^, the first linear regime is replaced by a second regime in which the foam height is fixed or at least increases more slowly (Figure 2b). This second regime was expected following Bikerman's work [62]. As bubbling is still performed at the same flow rate at this stage, this second regime is necessarily explained by enhanced bubble coalescence. It is reached sooner and for smaller foam heights for less concentrated microgel dispersions, as these foams initially contain less liquid and have larger bubbles, which in turn favor coalescence. It again proves that the foamability is reduced for smaller microgels concentrations.

We can notice in Figure S6 that foams generated with higher C_µG_ are less destabilized at τ = 16 min after their formation. This was expected considering that enhanced foamability was associated to an initial foam structure—smaller bubble size, higher liquid fraction, and probably larger surface coverage—promoting slow aging.

Overall, foamability increases with the microgel concentration, and the resulting foams exhibit larger liquid fractions, smaller bubble sizes, and undergo fewer coalescence events. The results suggest that faster adsorption of microgels at higher C_µG_ might account for earlier bubble detachment and higher surface coverage of bubbles, which would lead to smaller bubbles and enhanced foam stability. A more economical approach to tune foamability would be to modulate the microgel characteristics instead of increasing microgels concentration. Indeed, it has been reported that changes in microgel architecture can significantly impact foaming properties. Accordingly, we show that replacing the conventional BIS crosslinker with our supramolecular crosslinker drastically improves microgel foamability (Figure S7). Since this result may arise from differences in microgel size and/or internal structure, we disentangle these effects by investigating microgels with independently tuned size and structure. Note that these architectural changes are also expected to modify the microgels adsorption kinetics [18, 63].

### 2.4 Decoupling Effect of Microgel Size and Structure on the Adsorption Dynamics and Foamability

As shown in Table S1, two key microgel parameters have been independently varied: (i) the microgel size (µG‐R‐*xx* presented in empty symbols) has been tuned while keeping a fixed core‐shell structure and (ii) the microgel structure (µG‐CS‐*yy* presented in full symbols) has been tuned while keeping the microgel swollen size almost constant. All foaming experiments were conducted under identical conditions, with a fixed microgel concentration of 1 mg mL^−1^ in the region of low foamability on purpose, to clearly distinguish the influence of the microgel characteristics on foamability, drainage, and coalescence.

**FIGURE 3:**
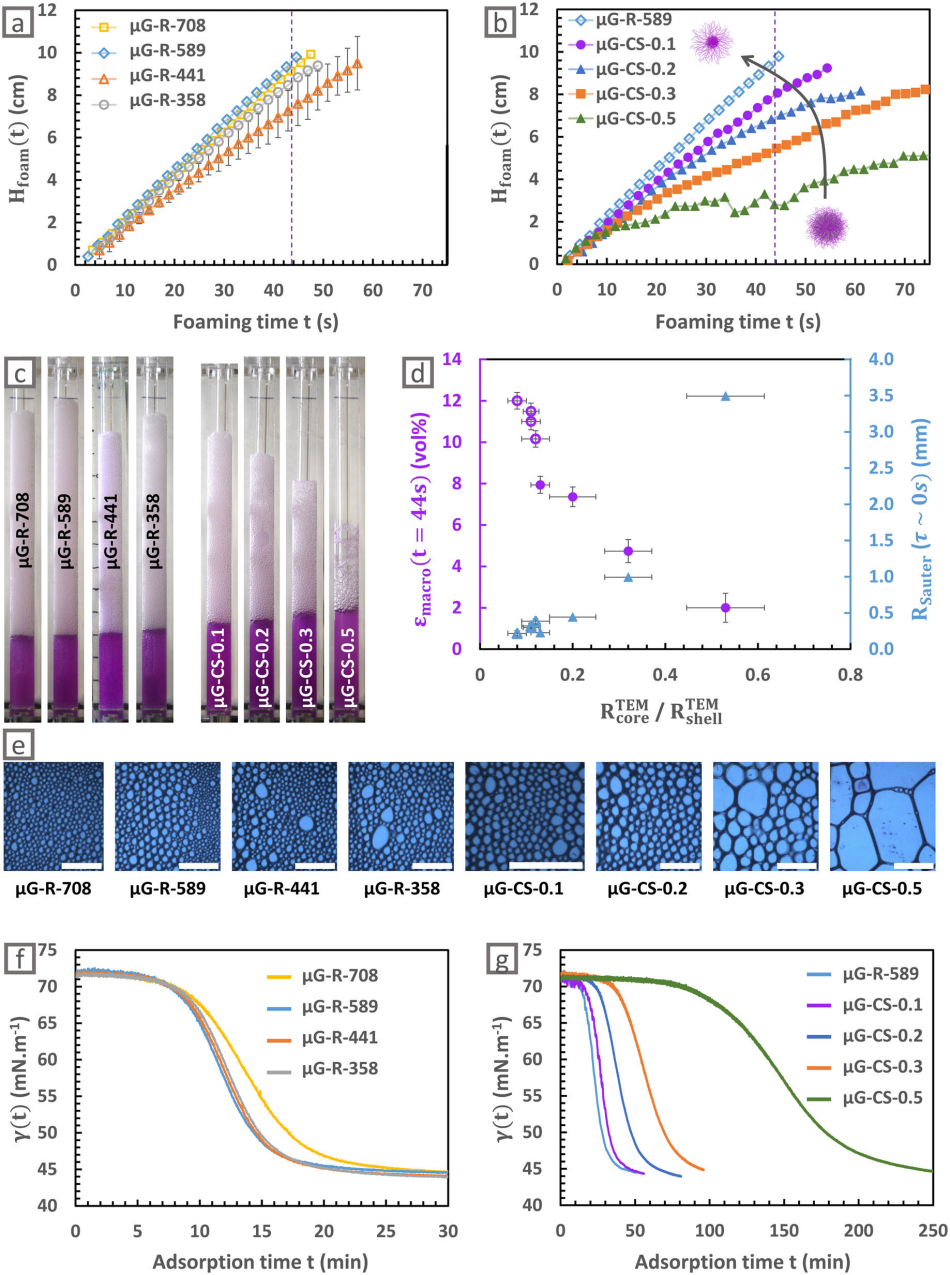
Effect of the microgel size and structure. The microgel concentration was fixed at 1 mg mL^−1^. Height of the foam (H_foam_(*t*)) as a function of the foaming time *t* for microgels of varying (a) size (empty symbols) and (b) structure (full symbols). Result for microgel µG‐R‐589 is represented in both graphs to ease the comparison. (c) Macroscopic images of the foams stabilized by microgels of independently tuned sizes and structures during foaming (at *t* = 44 s). (d) Average macroscopic liquid fraction ε_macro_ during foaming (at *t* = 44 s) and the average bubble radius R_Sauter_ at the end of the foaming process for microgels of varying size (empty symbols) and structure (full symbols) as a function of their core‐shell ratio $R_{\mathrm{core}}^{\mathrm{TEM}}/R_{\mathrm{shell}}^{\mathrm{TEM}}$. (e) Local images of the foam at the end of foaming for different microgels of independently tuned sizes and structures. The scale bar is always 5 mm. Spontaneous adsorption kinetics of the microgels from (f) varying size at 0.01 mg mL^−1^ and (g) varying structure at 0.005 mg mL^−1^ in pure water. Result for microgel µG‐R‐708 is represented in both graphs to ease comparison.

Figure 3a (resp. 3b) shows the evolution of the foam height H_foam_ as a function of the foaming time t for the microgels of varying size (resp. varying structure), and Figure 3c shows the macroscopic pictures of these foams taken at 44 s during foaming. For the microgels of varying size, the foam heights in Figure 3c are almost similar, and no clear trend is observed when tuning the microgel size. By contrast, for the microgels of varying structure, the foam height clearly decreases, and the liquid height slightly increases for more homogeneous structures, already indicating that more homogeneous structures are less efficient to stabilize foams.

More precisely, the foam heights presented in Figure 3a superimpose well. Thus, changing the microgel size does not seem to significantly impact the foamability. Furthermore, as shown in Figure 3d, small bubbles with similar R_Sauter_ between 0.2 and 0.3 mm are formed, no matter the microgel size, which can be correlated to the similar microgel adsorption kinetics presented in Figure 3f. It can be noticed that microgel adsorption is not led by microgel diffusion, as it was already concluded earlier [26], since in our case the microgel size does not affect the adsorption kinetics. In addition, similar high liquid fractions ε_macro_ between 10 and 12 vol% are obtained for microgels with varying sizes, as reported in empty symbols in Figure 3d. Finally, microgel size does not drastically modify the foam destabilization kinetics as proven by the similar macroscopic images of the foams during destabilization presented in Figure S8. To conclude, neither the foamability nor the foam stability are significantly affected by the microgel size for such “ultra” core‐shell structures.

On the contrary, Figure 3b shows that for more homogeneous microgels such as µG‐CS‐0.5, the foam height increases more slowly with some erratic drops, indicating catastrophic bubble coalescence during foam formation. As already discussed with Figure 2b, it results in a transition from a linear regime to a second regime with enhanced bubble coalescence. This transition occurs for shorter foaming times and smaller foam heights in the case of more homogeneous microgels, proving again their lower foamability. Consistently, the average bubble size R_Sauter_ drastically increases from 0.2 to 3.5 mm when the core to (core + shell) radius ratio increases from 0.1 to 0.5, as indicated in Figure 3d,e. Similar to the discussion on the role of concentration, we suggest that the increase in bubble size for more homogeneous structures are correlated to the slower microgel adsorption kinetics shown in Figure 3g. The slower adsorption kinetics can be explained by the reduction of dangling chains when distributing more homogeneously the crosslinkers. Indeed, it has been suggested in literature that these dangling chains play a key role in the collective adsorption of the microgels at the interface [18]. It could also be explained by the larger electrophoretic mobility of the homogeneous microgels (Table S1), indicating higher surface charge density. Electrostatic repulsion between the microgels is indeed expected to slow down their adsorption [18, 22, 64].

Furthermore, the liquid fraction ε_macro_ measured at t = 44 s decreases from 12 to 2 vol% when the core to (core + shell) radius ratio increases from 0.1 to 0.5. This result can be linked to faster drainage for more homogeneous microgels. First, as mentioned before, drainage is accelerated in the presence of larger bubbles. Second, the reduction of dangling chains and the vanishing of the shell, due to a more homogeneous distribution of the crosslinkers, limits the interactions between microgels. Yet, these interactions are probably necessary to locally increase the liquid viscosity in the Plateau borders, so that reduced interactions would result in a lower ability of more homogeneous microgels to limit drainage.

Thus, more homogeneous microgels are less efficient to limit both drainage and coalescence events, which reduces the obtained foam heights. In addition, all these results also coincide with the lower stability of the foams generated with more homogeneous microgels, as indicated by the macroscopic images of the destabilizing foams in Figure S8.

To conclude, the microgel structure is a key parameter governing the foamability, while the microgel size has no significant effect. We mainly correlated the remarkably enhanced foamability of “ultra” core‐shell microgels with faster microgel adsorption kinetics. Indeed, a faster adsorption might lead to reduced bubble sizes and enhanced surface coverage, which both slow down drainage and reduce coalescence events.

In addition to the concentration and structure of stabilizers, the adsorption kinetics and surface coverage at short times during foaming can also be controlled by tuning the ionic strength [18].

### 2.5 Salt Addition to Accelerate the Adsorption Kinetics and Hence Improve the Foamability

**FIGURE 4:**
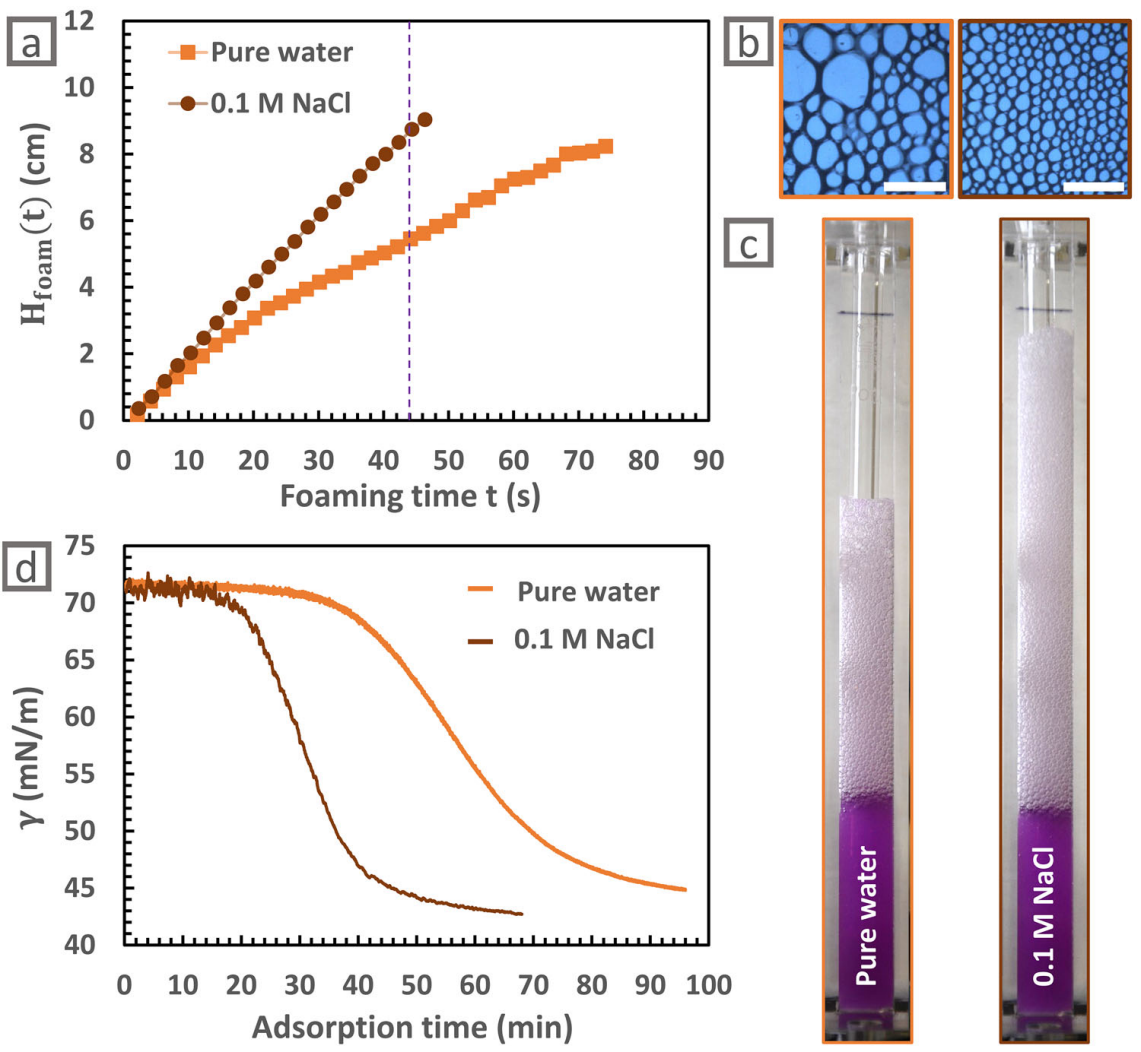
Impact of salt addition on foamability of µG‐CS‐0.3. (a) Height of the formed foam (H_foam_(*t*)) as a function of the foaming time *t* for µG‐CS‐0.3 dispersed at 1 mg mL^−1^ either in pure water or in 0.1 mol L^−1^ NaCl. (b) Local images at the end of the foaming process. Scale bar is 5 mm. (c) Macroscopic pictures during the foaming at *t* = 44 s for foams formed from µG‐CS‐0.3 dispersions either in pure water or in 0.1 mol L^−1^ NaCl. (d) Spontaneous adsorption kinetics of the µG‐CS‐0.3 at 0.005 mg mL^−1^ in pure water and in 0.1 mol L^−1^ NaCl.

According to our previous hypothesis, accelerating the adsorption kinetics of the microgels is a good way to increase the foamability of a microgel dispersion. An easy way to tune the adsorption kinetics for charged microgels is to increase the ionic strength in the medium by adding salt. Indeed, Tatry et al. explain the faster microgel adsorption kinetics measured in the presence of salt by the screening of electrostatic interactions responsible for an adsorption barrier [18]. Thus, the influence of salt addition on foamability is presented in this last section (Figure 4).

Consistently, Figure 4a,c show that larger foam heights are reached when foaming the same microgel dispersion in the presence of salt. Similarly, Table 1 shows a tremendous reduction of air loss during the foaming in salty conditions (only 6 % in 0.1 mol L^−1^ NaCl against 41 % in pure water), which confirms the reduction of coalescence events. Besides, smaller bubble sizes are obtained when adding salt in the foaming dispersion (Figure 4b and Table 1). All these results can again be correlated to a faster microgel adsorption (Figure 4d).

**TABLE 1:** Characteristics of foams formed with µG‐CF13 at 1 mg mL^−1^ either in pure water or in salt.

	R_Sauter_ (mm)	ε_macro_(t = 44 s) (%v)	Air loss fraction (%)
Pure water	1.0	5 ± 1	41 ± 2
NaCl 0.1 M	0.4	5 ± 1	6 ± 2

Consistent with the above results, foam destabilization is slowed down when foam is generated in the presence of salt (Figure S9). Such behavior was already observed in the literature for foams stabilized by silica particles [65]. In our case, it is to be correlated with the foam initial structure, containing smaller bubbles which slow down the drainage and limit the aging of the foam.

## 3 Conclusions

In this article, Pickering foams stabilized by microgels were formed by bubbling air into supramolecular microgel dispersions. Compared with the commonly used Bartsch method, this bubbling approach enables direct monitoring of the foamability and rapid production of large foam volumes (more than 20 cm^3^ in less than 45 s) that remain stable for several hours. For the first time, a systematic study of the influence of microgel size and structure on the foamability is performed to identify the key parameters determining the foam properties. No significant effect of the microgel size for core‐shell microgels is observed, while both an increase of the microgel concentration or an increase in the core‐shell structure lead to an enhanced foamability, a decrease in bubble size and an increase in liquid fraction. These changes in the foam initial structure result in turn in an improved foam stability. We propose a link between these observations and the microgel adsorption kinetics: faster microgel adsorption kinetics lead to an enhanced foamability. Such kinetics can be accelerated by increasing the microgel concentration, screening electrostatic repulsions by adding salt, using more core‐shell structures, but not by tuning the microgel size. Overall, the study establishes a clear link between the microgel architecture and foam properties, paving the way for new microgel‐stabilized Pickering foams.

## Conflicts of Interest

The authors declare no conflicts of interest.

## Supporting information




**Supporting File**: smll72432‐sup‐0001‐SuppMat.docx.

## Data Availability

The data that support the findings of this study are available from the corresponding author upon reasonable request.
