# Supplementary material for: How PNIPAM Microgel Architecture Controls Pickering Foam Formation
Source: Small. 2026 Feb 2;22(15):e13819. doi: 10.1002/smll.202513819 (PMC12980458; doi:10.1002/smll.202513819)
Supplement: Supplementary file 1 — Supporting File: smll72432‐sup‐0001‐SuppMat.docx. [file SMLL-22-e13819-s001.docx]

Supporting Information

How PNIPAM Microgel Architecture Controls Pickering Foam Formation

Antoine Brézault^1,2,3 +^, Anne R. Rousseau^3,4,5 +^*, Véronique Schmitt^1^, Valérie Ravaine^2^, Patrick Perrin^3^, Nicolas Sanson^3^*, Cécile Monteux^3^

^1^ Centre de Recherche Paul Pascal, Université de Bordeaux, CNRS UMR 5031, 115 Avenue Dr Albert Schweitzer, 33600 Pessac, France

^2^ Bordeaux INP, ISM, Université de Bordeaux, CNRS UMR 5255, 16 Avenue Pey Berland, 33400 Talence, France

^3^ Soft Matter Sciences and Engineering, ESPCI, PSL University, Sorbonne Université, CNRS UMR 7615, 10 rue Vauquelin, 75231 Paris Cedex 05, France.

^4^ Institut de Recherche de Chimie Paris (IRCP), Chimie ParisTech, PSL Research University, CNRS, Paris 75005, France

^5^ Agence de l’Environnement et de la Maîtrise de l’Energie (ADEME), 20 avenue du Grésillé, 49004 Angers, France

^+^ Co-first authors

*corresponding author

Table of content

[I. Experimental section 2](#_Toc216359090)

[I.1 Microgels synthesis 2](#_Toc216359091)

[I.3. Foam formation and characterization 5](#_Toc216359092)

[*Set-up* 5](#_Toc216359093)

[*Foaming dispersions* 5](#_Toc216359094)

[*Foaming process* 6](#_Toc216359095)

[*Pictures acquisition* 6](#_Toc216359096)

[*Data analysis* 6](#_Toc216359097)

[II. Supplementary data 7](#_Toc216359098)

[II.1. Microgel characterization 8](#_Toc216359099)

[II.2. Microgels purification 10](#_Toc216359100)

[II.3. Time evolution of local liquid fraction 11](#_Toc216359101)

[II.4. Foam and liquid height increase rates for different microgel concentrations 12](#_Toc216359102)

[II.5. Adsorption kinetics for different microgel concentrations 12](#_Toc216359103)

[II.6. Foam stability for different microgel concentrations 13](#_Toc216359104)

[II.8. Foam stability microgels of independently tuned sizes and structures 15](#_Toc216359105)

[II.9. Foam stability in salty conditions 16](#_Toc216359106)

[III. References 17](#_Toc216359107)

# Experimental section

## I.1 Microgels synthesis

Supramolecular microgels were synthesized by copolymerizing a homemade supramolecular crosslinker (SC) with N-isopropylacrylamide (NIPAM) as the main monomer, using classical polymerization in dispersion, as described in previous studies ^[1,2]^. The SC is based on a Fe(II)/terpyridines complex (Figure S0b) . Two series of microgels were synthesized with the objective to independently tune the microgel size and the microgel crosslinker distribution (i.e., microgel structure), keeping the crosslinker content close to 1 mol% *vs* NIPAM (Figure S0). The conditions and amount of reactants used for each microgel synthesis are described in detail in Figure S0.


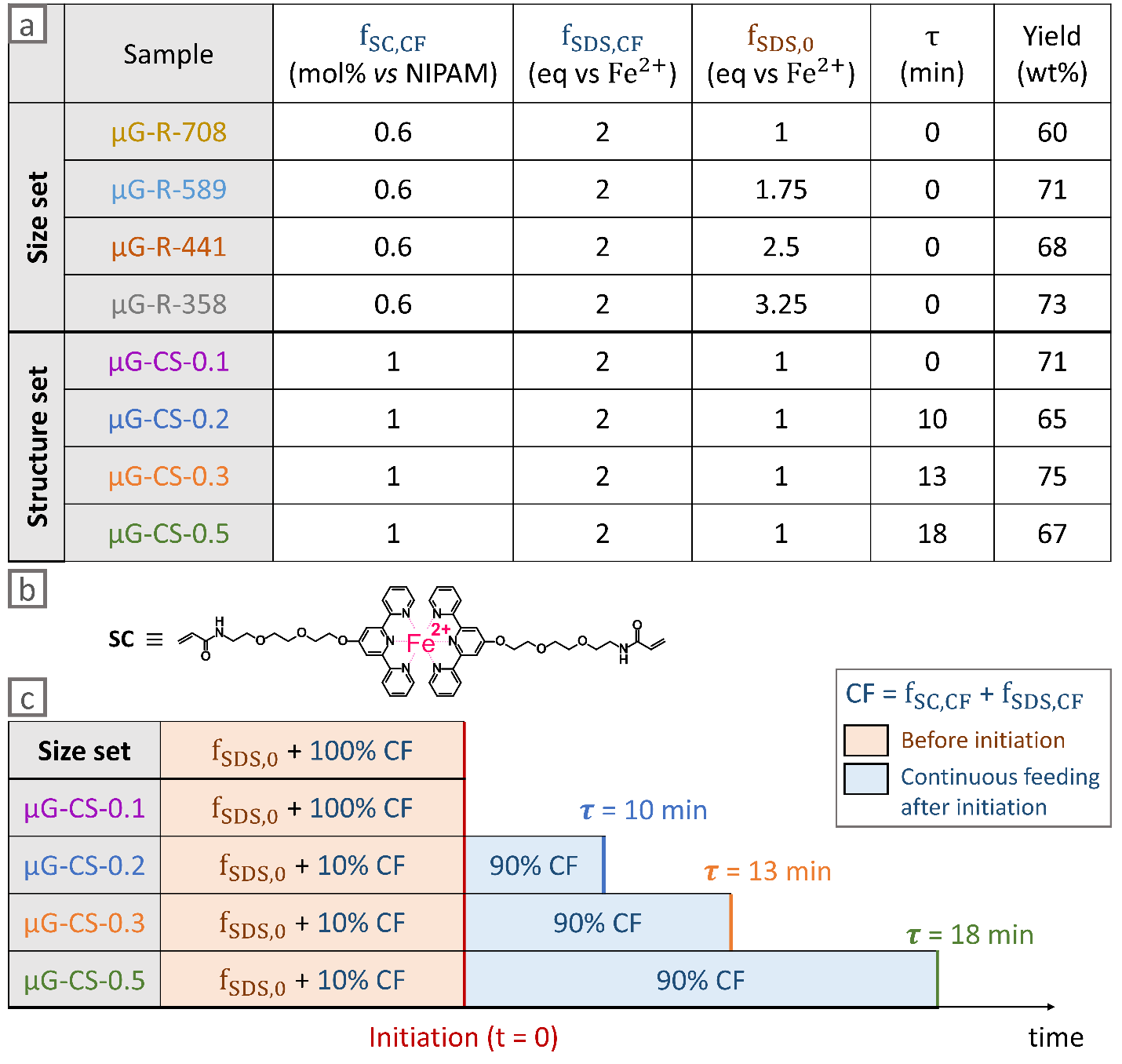


**Figure S0.** Synthesis conditions for the microgels from the size and structure sets. a) reactants content and c) feeding method used for the syntheses. b) topological view of the supramolecular crosslinker (SC) used in all the syntheses.

Briefly, the monomer NIPAM (always 1 wt% of the total reaction mixture) and sodium dodecyl sulfate (SDS, $f_{SDS,0}$) were dissolved in purged water. The home-made SC ($f_{SC,CF}$), synthesized as reported in ^[3]^, and additional SDS ($f_{SDS,CF}$, 2 eq *versus* Fe^2+^) were mixed together in an external vial. This solution, called SC solution, was added; either directly in the flask before initiation, for microgels of “Size” set; or progressively during a certain time after initiation, for microgels from the “Structure set” synthesized with a continuous feeding method. Thus, microgels µG-R-358, µG-R-441, µG-R-589 and µG-R-708 were synthesized in batch using respectively 5.25, 4.5, 3.75 and 3 eq. of SDS *vs* SC. More detailed information on the synthesis process of microgels of controlled size can be found in reference ^[1]^. Microgels µG-CS-0.1, µG-CS-0.2, µG-CS-0.3 and µG-CS-0.5 were synthesized adding the SC solution before initiation and/or during 10, 13 or 18 min. More detailed information on the synthesis process of microgels of controlled structure can be found in reference ^[2]^.

After initiation by V50 (2 mol% *versus* NIPAM), the polymerization reactions were allowed to proceed for 4 h at 70 °C. Then, the mixtures were cooled down to room temperature and purified by dialysis. After the synthesis, the microgel dispersions were purified by dialysis (Biotech CE 1,000,000 MWCO) for more than 10 days in water to remove oligomers, unreacted monomers, and surfactant molecules. During this time, water was changed twice a day. After the purification process, the microgels were freeze-dried and dispersed in water at the desired concentration before using. The microgels characteristics are reported in Figure S0.

## I.3. Foam formation and characterization

### Set-up

A homemade rectangular column of internal section 1 cm x 2 cm and of height 14.5 cm was designed in polymethylmethacrylate (PMMA). A right-angle prism (Edmund Optics) was glued at 6.5 cm from the bottom of the column with a drop of a mixture of 0.05 mol.L^-1^ BrijO10 and 0.1 mol.L^-1^ glycerol to ensure optical contact. A LED light panel was used to illuminate the column in a plane parallel to one free face of the prism, while a camera was placed parallel to the other side of the right angle prism to acquire the foam images. This set-up allows probing the first layer of bubbles in contact with the column wall. It ensures a contrast between the foam Plateau borders, which appear dark because their curves refract light, and the films, which appear brighter because their flat area reflects light ^[4,5]^.

### Foaming dispersions

The foaming dispersions were prepared by dispersing a known mass of freeze-dried microgels in water. They were agitated during at least one night using a reciprocating shaker to ensure proper dispersion of microgels, and were used afterwards without further preparation.

### Foaming process

For each experiment, 8 mL of foaming dispersion were introduced in the column. A porous frit (ROBU® micro filter candle, pore size = 16 – 40 µm) was then immersed in the liquid for 1 minute ensuring no flow thanks to a mass flow controller (MKS, model GE50A013102SMV020, powered by MKS PR4000BS2V2 power supply). Afterwards, an air flow rate of 20 mL.min^-1^ was forced through the porous frit using the mass flow controller, which produced bubbles. The air flow was stopped once the total height of liquid and foam reached 10 cm.

### Pictures acquisition

Pictures of the whole column were taken every 2 seconds during the foaming process by a fixed Nikon camera (camera D7000 equipped with Nikon AF-S DX Nikkor 18-105mm f/3.5-5.6G ED VR objective).

Pictures of the bubbles at the wall were taken every 5 seconds at the level of the prism (about 6.5 cm from the column bottom) using a PixeLink CMOS Camera PL-B953U equipped with a telecentric objective.

### Data analysis

The pictures acquired with the prism are deformed by a $\sqrt{2}$factor for geometrical reasons. Pictures were consequently rescaled before being binarized using a Python program. The surface area of each bubble was determined from the binarized images and converted into the bubble radius $R_{b}= \sqrt{A_{b}/\pi}$, which is the radius of the sphere having the same area as the considered bubble. This method is widespread in the literature and it is admitted that the average areal bubble radius is consistent with average volume bubble radius ^[6–9]^. The average Sauter radius $R_{\mathrm{Sauter}}= \frac{\sum{R_{b}}^{3}}{\sum{R_{b}}^{2}}$ was calculated and used to compare the mean bubble size in various foams, as recommended in the case of polydisperse distributions ^[10,11]^.

After about 4 seconds, foam height H_foam_(t) grows linearly with time, while the liquid height H_liquid_(t) in the liquid reservoir below the foam decreases linearly with time. As the time of beginning of bubbling is known with a precision of 2 seconds, data were shifted in time so that the linear model goes through (0;0) to accurately set the zero time.

# Supplementary data

## II.1. Microgel characterization


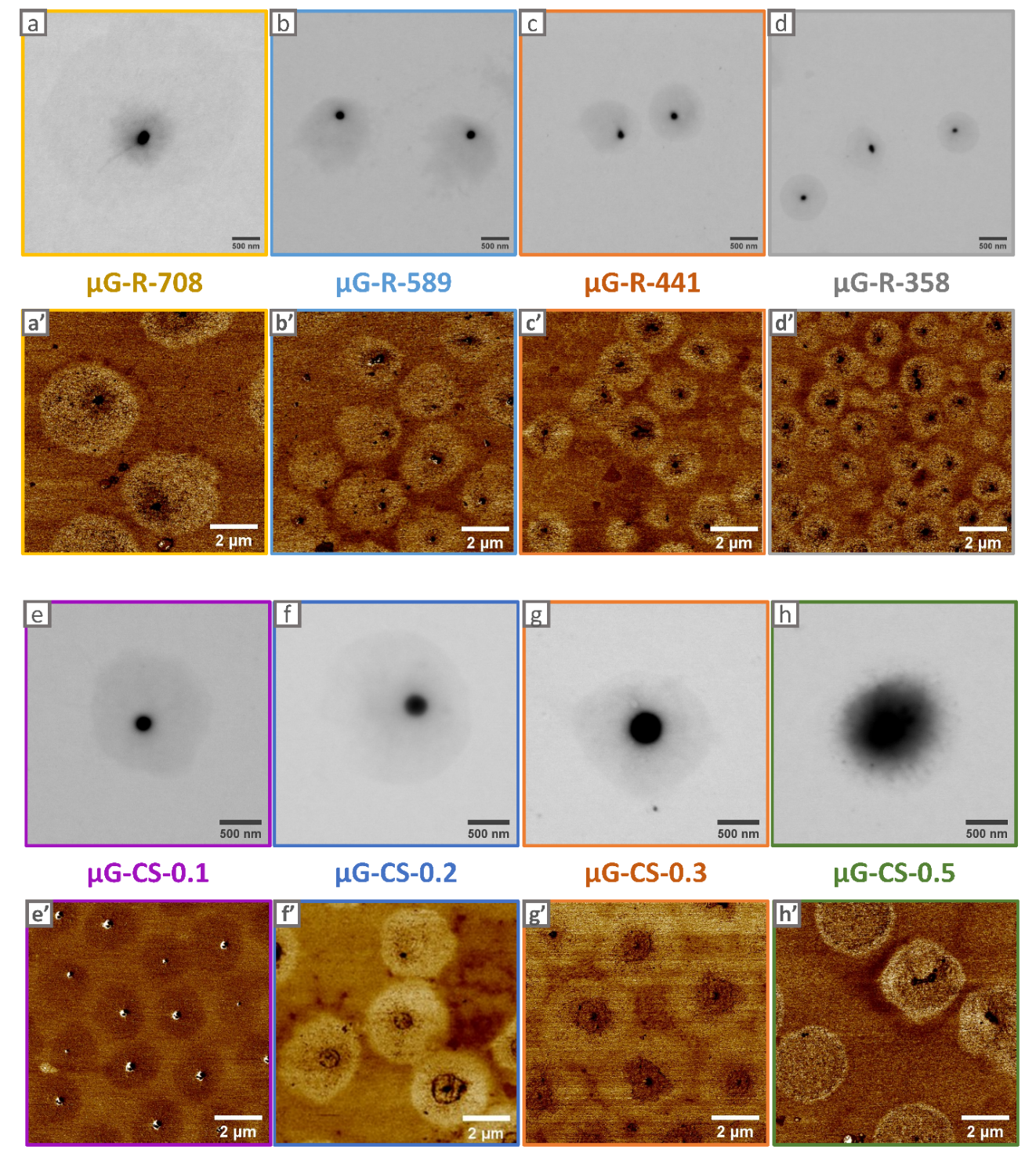


**Figure S1.** TEM (letter) and phase AFM (letter’) images of dried microgels from various sizes (a-d) and from various structures (e-i). For AFM samples, microgels were transferred from a Langmuir film at zero pressure.

**Table S1.** Characteristics of the supramolecular microgels synthesized in order to vary either their hydrodynamic radius (size variation) or their core to (core + shell) ratio (structure variation).

|  | **Size variation** | | | | **Structure variation** | | | |
| --- | --- | --- | --- | --- | --- | --- | --- | --- |
| **Sample** | **µG**  **-R-358** | **µG**  **-R-441** | **µG**  **-R-589** | **µG**  **-R-708** | **µG**  **-CS-0.1** | **µG**  **-CS-0.2** | **µG**  **-CS-0.3** | **µG**  **-CS-0.5** |
| Supramolecular crosslinker ratio **r(SC)**  **(%mol vs NIPAM)** | 0.86  ± 0.02 | 0.91  ± 0.02 | 0.99  ± 0.02 | 0.60  ± 0.02 | 1.02  ± 0.02 | 1.05  ± 0.02 | 1.12  ± 0.02 | 0.95  ± 0.02 |
| Hydrodynamic radius $\mathbf{R}_{\mathbf{H}}\left( \boldsymbol{20 ^{\circ}C} \right)$  **(nm)** | **358**  ± 6 | **441**  ± 8 | **589**  ± 7 | **708**  ± 18 | 589  ± 10 | 665  ± 15 | 568  ± 9 | 577  ± 5 |
| Hydrodynamic radius $\mathbf{R}_{\mathbf{H}}\left( \boldsymbol{70 ^{\circ}C} \right)$  **(nm)** | 97  ± 2 | 123  ± 2 | 144  ± 2 | 185  ± 5 | 157  ± 2 | 179  ± 5 | 165  ± 8 | 207  ± 9 |
| Electrophoretic mobility $\boldsymbol{\mu(20 ^{\circ}C)}$  **(µ**$\mathbf{m.cm.}\mathbf{V}^{\mathbf{-1}}\mathbf{.}\mathbf{s}^{\mathbf{-1}}$**)** | 0.58  ± 0.02 | 0.60  ± 0.01 | 0.50  ± 0.02 | 0.46  ± 0.03 | 0.63  ± 0.02 | 0.61  ± 0.04 | 0.84  ± 0.02 | 1.24  ± 0.04 |
| CS size ratio assessed by TEM  $\mathbf{R}_{\mathbf{core}}^{\mathbf{TEM}}$ **/** $\mathbf{R}_{\mathbf{shell}}^{\mathbf{TEM}}$ | 0.12  ± 0.03 | 0.11  ± 0.02 | 0.11  ± 0.03 | 0.08  ± 0.02 | **0.13**  ± 0.02 | **0.20**  ± 0.04 | **0.32**  ± 0.04 | **0.53**  **± 0.08** |
| CS size ratio assessed by AFM  $\mathbf{R}_{\mathbf{core}}^{\mathbf{AFM}}$ **/** $\mathbf{R}_{\mathbf{shell}}^{\mathbf{AFM}}$ | 0.12  ± 0.03 | 0.12  ± 0.02 | 0.11  ± 0.02 | 0.10  ± 0.01 | 0.14  ± 0.02 | 0.24  ± 0.09 | 0.42  ± 0.06 | - |

Crosslinker content r(SC) was evaluated by UV-Visible spectroscopy. Microgels swollen $R_{H}(20 ^{\circ}C)$ and collapsed $R_{H}(70 ^{\circ}C)$ hydrodynamic radii were evaluated by multiple angle light scattering. $R_{\mathrm{core}}^{\mathrm{TEM}}/R_{\mathrm{shell}}^{\mathrm{TEM}}$ and $R_{\mathrm{core}}^{\mathrm{AFM}}/R_{\mathrm{shell}}^{\mathrm{AFM}}$ are measurements of the size ratios between the microgels core and its (core + shell). These sizes were measured respectively from images obtained by Transmission Electron Microscopy (TEM) or Atomic Force Microscopy (AFM). These are mean values coming from the measurements on approximately 60 microgels from different images similar to those presented in Figure S1. Electrophoretic mobility $\mu(20 ^{\circ}C)$ was evaluated on a Zetasizer Nano-ZS93 (Malvern) device in the presence of NaCl 0.1 mM.

## II.2. Microgels purification

Comparing the adsorption kinetics of the microgels dispersion with the ones from the associated supernatant after centrifugation (Figure S2) proves that the former is prevalent. After the purification process, the microgels were freeze-dried for storage.


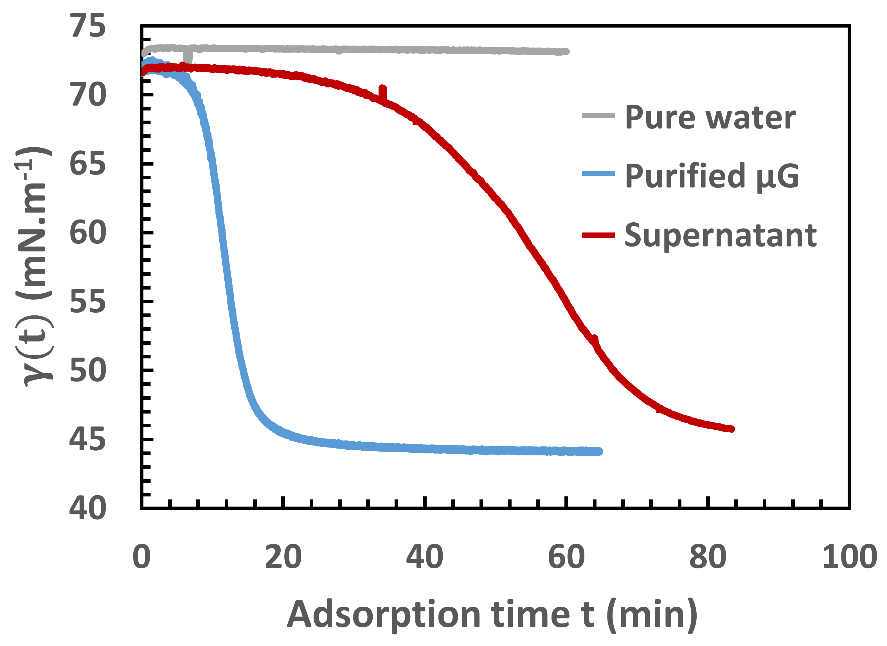


**Figure S2.** Adsorption kinetics measured by pendant drop tensiometry for pure water, dispersion at a 0.01 mg.mL^-1^ concentration of µG-R-589 purified by dialysis, and supernatant of a centrifuged dispersion of purified µG-R-589 at 0.01 mg.mL^-1^.

## II.3. Time evolution of local liquid fraction


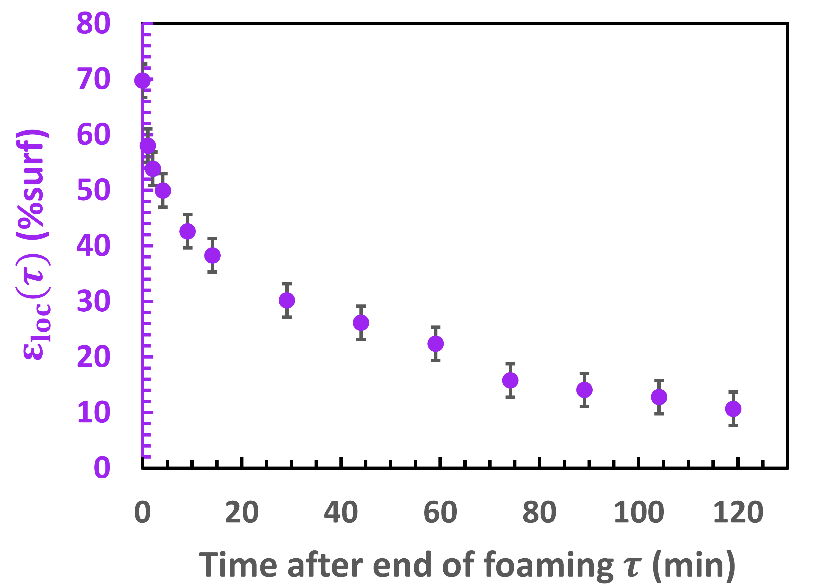


**Figure S3.** Local liquid fraction $\varepsilon_{\mathrm{loc}}$ measured as a function of time after end of foaming thanks to the local images presented in the main document in Figure 1e.

We chose not to convert $\varepsilon_{\mathrm{loc}}$ into a volume fraction since the existing formula has been established for non-adhesive and non-elastic systems ^[12,13]^, which might likely not be the case with interface stabilized by microgels. Moreover, $\varepsilon_{\mathrm{loc}}$ gives a local insight of the foam, whereas $\varepsilon_{\mathrm{macro}}$ is the result of a mean on the whole height of foam. A deeper study to build a general link between these two measurements is on-going.

## II.4. Foam and liquid height increase rates for different microgel concentrations


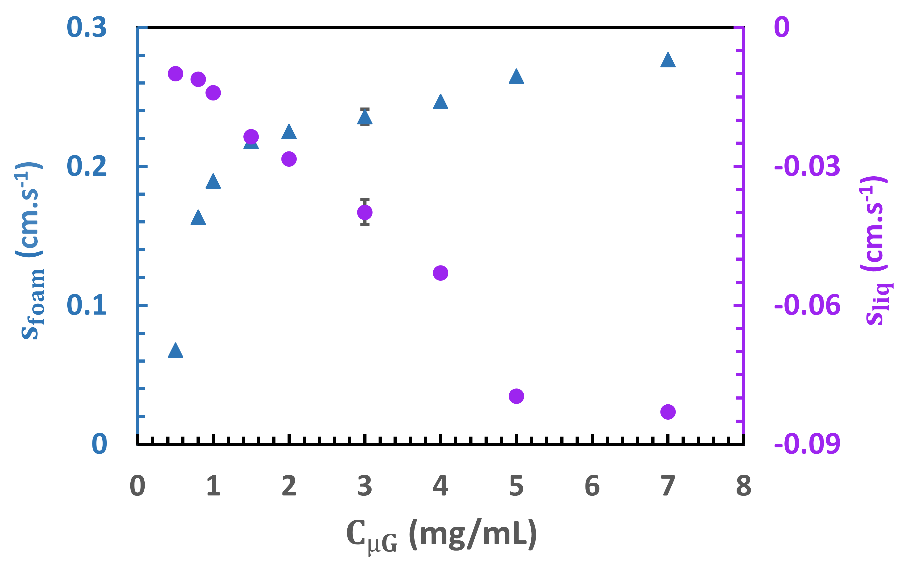


**Figure S4.** Foam ($s_{\mathrm{foam}}$) and liquid ($s_{\mathrm{liq}}$) height increase rates defined as the slopes on Figure 2b and 2c, when bubbling in a microgel dispersion at different concentrations $C_{\mu G}$. Error bars represented for $C_{\mu G}=3$ mg.mL^-1^ have been calculated from the 4 experiments represented in Figure 1b and 1d.

## II.5. Adsorption kinetics for different microgel concentrations


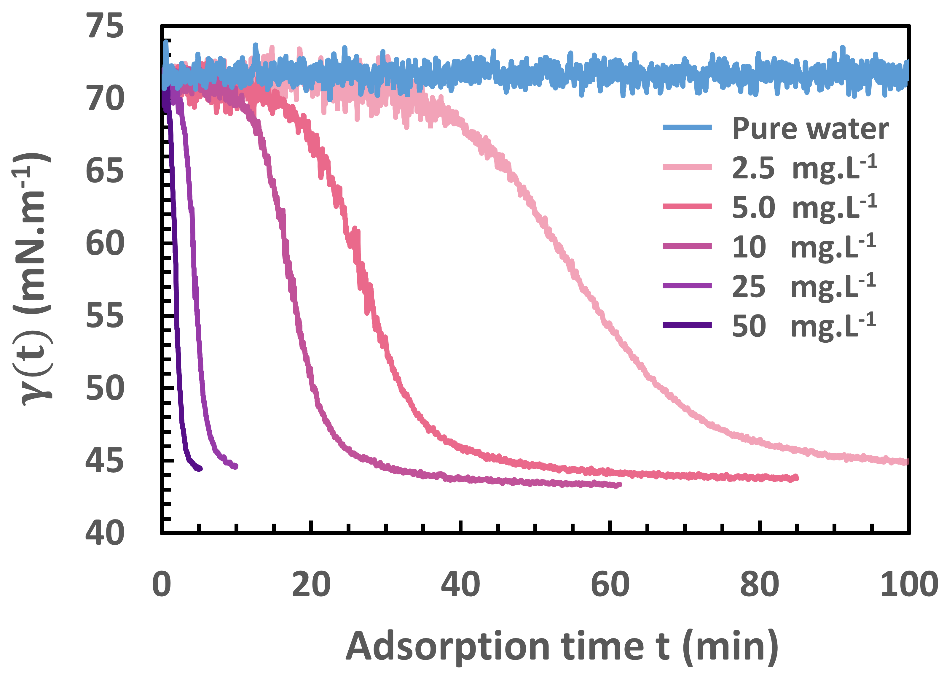


**Figure S5.** Adsorption kinetics measured by pendant drop tensiometry for µG-CS-0.1 dispersed in pure water at different concentrations.

## II.6. Foam stability for different microgel concentrations


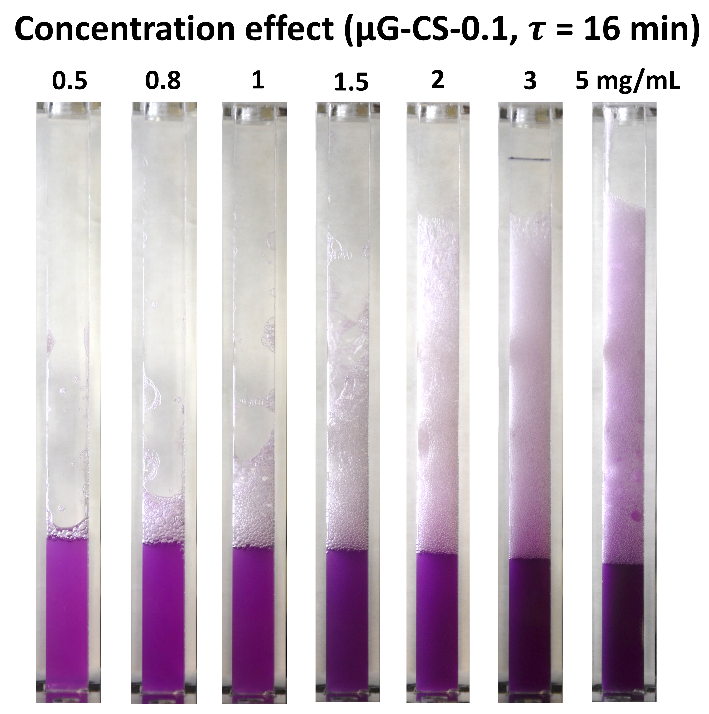


**Figure S6.** Macroscopic images of the foams stabilized by different concentrations of µG-CS-0.1 in mg.mL^-1^, 16 minutes after the end of foaming. Scale is given by the column internal width which measures 10 mm.

II.7. Compared foamability for supramolecular and classical PNIPAM-BIS microgels
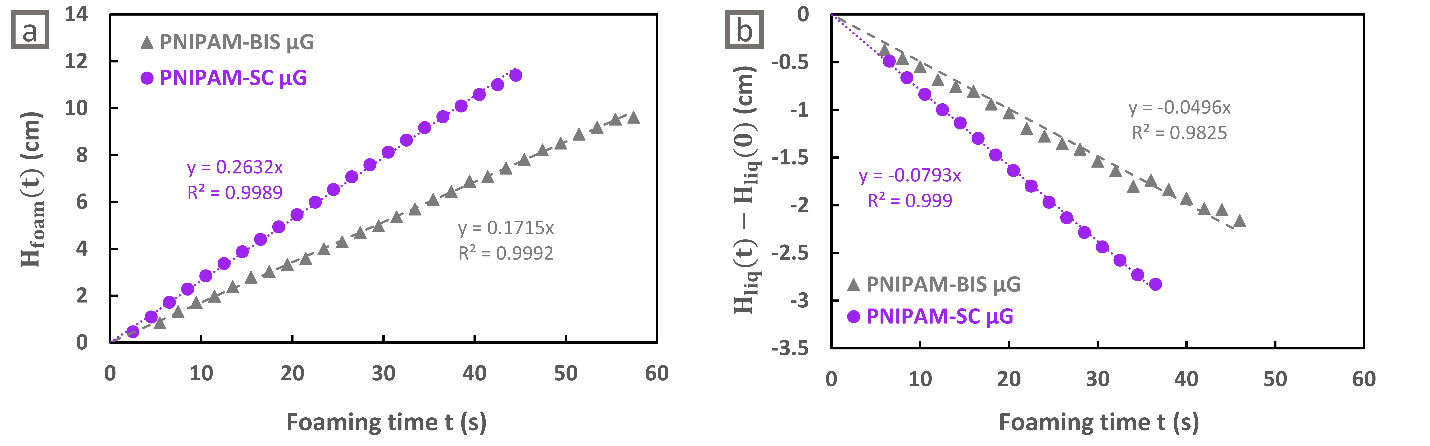


**Figure S7.** a) Height of the foam during foaming ($H_{\mathrm{foam}}(t)$) and b) difference between the liquid height at times t and t = 0 ($H_{\mathrm{liq}}(t)-H_{\mathrm{liq}}(0)$) as a function of the foaming time t for 5 mg/mL dispersions of microgels synthesized in batch either with SC or BIS crosslinkers. The supramolecular microgel is the µG-CS-0.1 which characteristics are reported in the main text. The PNIPAM-BIS microgel has been synthesized without any surfactants with 2 mol% BIS vs NIPAM with the protocol reported in the Experimental Section. Its swollen size is $R_{H}\left( 20 ^{\circ}C \right)=360\pm10 nm$.

From the slopes obtained with the linear modeling of foam and liquid height evolutions with time, we calculate that the air flowrate effectively imprisoned in the foam is 12 mL/min for the PNIPAM-BIS microgel, whereas it reaches 19 mL/min for the PNIPAM-SC microgel. Thus, coalescence events are a lot more frequent during the foaming for the PNIPAM-BIS microgel. Note that the initial liquid fraction is similar in both cases: about 29 vol% for the PNIPAM-BIS microgel and 30 vol% for the PNIPAM-SC microgel.

## II.8. Foam stability for microgels of independently tuned sizes and structures


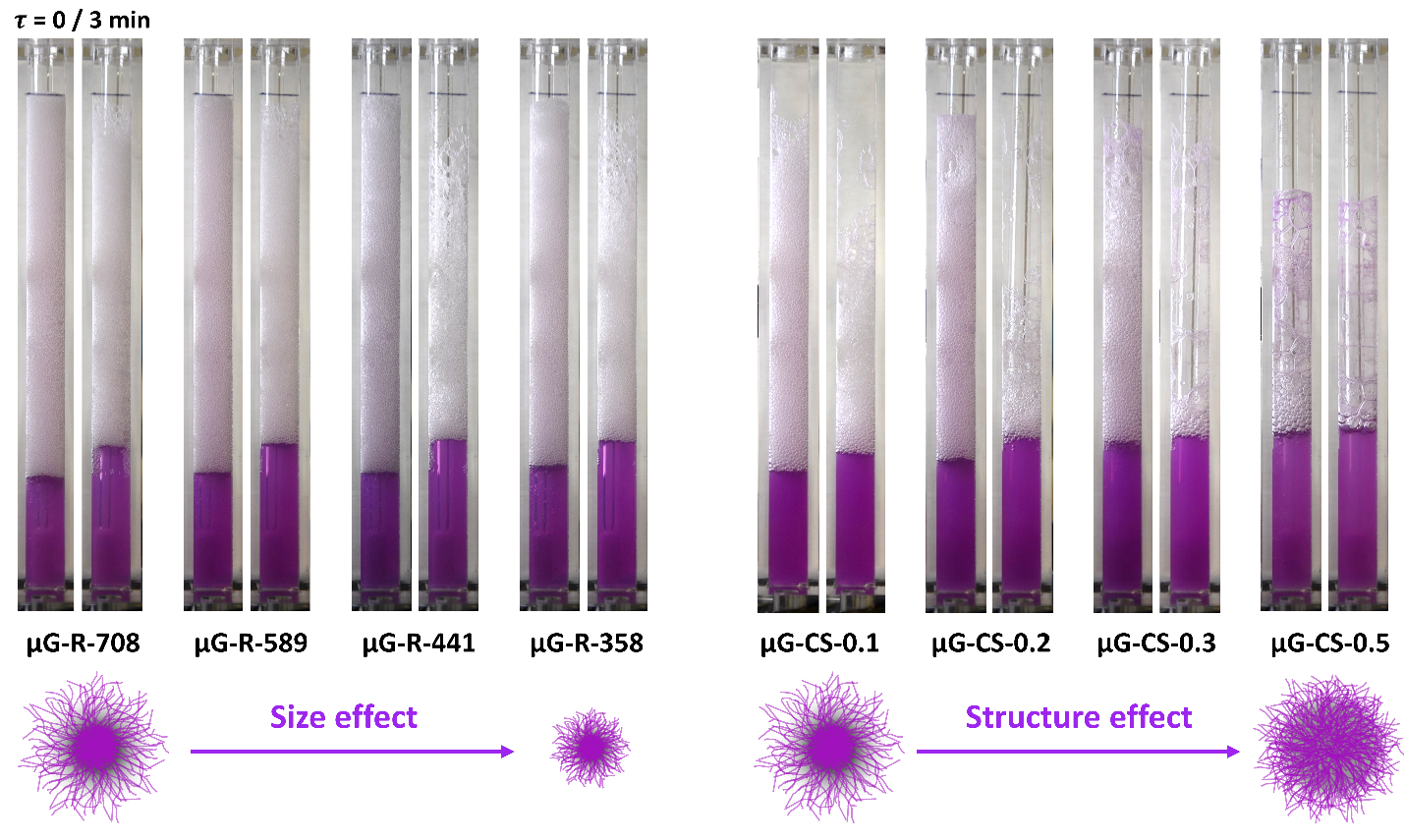


**Figure S8.** Macroscopic images of the foams stabilized by microgels from both sets at 1 mg.mL^‑1^, at the end of foaming and 3 minutes after the end of foaming. Scale is given by the column internal width which measures 10 mm.

## II.9. Foam stability in salty conditions


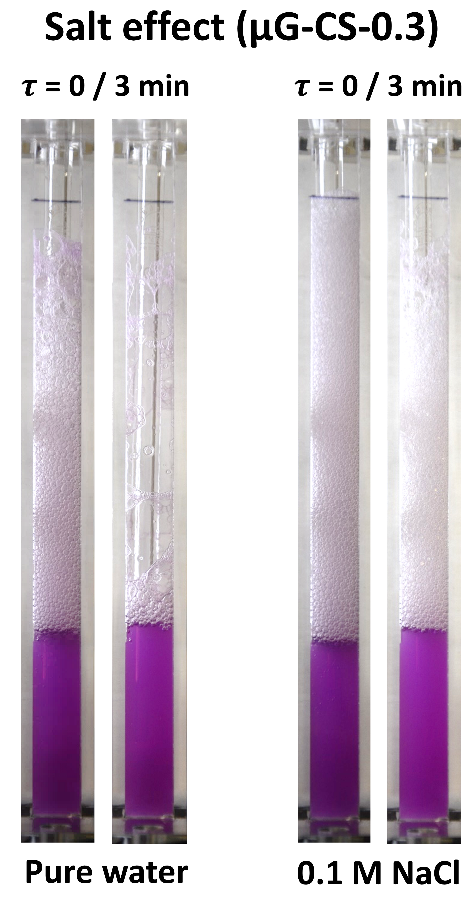


**Figure S9.** Macroscopic images of the foams stabilized by µG-CS-0.3 at 1 mg.mL^-1^, at the end of foaming and 3 minutes after the end of foaming. Microgels were dispersed either in pure water or in 0.1 mol.L^-1^ of NaCl. Scale is given by the column internal width which measures 10 mm.

# References

[1] A. Brézault, P. Perrin, N. Sanson, “Multiresponsive Supramolecular Poly(N-isopropylacrylamide) Microgels” *Macromolecules* **2024**, *57*, 2651–2660.

[2] A. Brézault, M. Hanafi, V. Schmitt, V. Ravaine, P. Perrin, N. Sanson, “Responsive properties and triggered disassembly of supramolecular microgels: a key role of the structure” *Polymer* **2025**, *336*, 128850.

[3] S. Laquerbe, J. Es Sayed, C. Lorthioir, C. Meyer, T. Narita, G. Ducouret, P. Perrin, N. Sanson, “Supramolecular Crosslinked Hydrogels: Similarities and Differences with Chemically Crosslinked Hydrogels” *Macromolecules* **2023**, *56*, 7406–7418.

[4] P. R. Garrett, J. D. Hines, S. C. Joyce, P. T. Whittal, *Report prepared for Unilever R&D*, Unilever R&D, **1993**.

[5] S. Mukherjee, H. Wiedersich, “Morphological and viscoelastic properties of dense foams generated from skin cleansing bars” *Colloids and Surfaces A: Physicochemical and Engineering Aspects* **1995**, *95*, 159–172.

[6] H. C. Cheng, R. Lemlich, “Errors in the measurement of bubble size distribution in foam” *Ind. Eng. Chem. Fund.* **1983**, *22*, 105–109.

[7] Y. Wang, S. J. Neethling, “Simulating realistic froth surfaces” *Minerals Engineering* **2006**, *19*, 1069–1076.

[8] Y. Wang, S. J. Neethling, “The relationship between the surface and internal structure of dry foam” *Colloids and Surfaces A: Physicochemical and Engineering Aspects* **2009**, *339*, 73–81.

[9] M. Pasquet, N. Galvani, O. Pitois, S. Cohen-Addad, R. Höhler, A. T. Chieco, S. Dillavou, J. M. Hanlan, D. J. Durian, E. Rio, A. Salonen, D. Langevin, **2023**, arXiv preprint, DOI: 10.48550/arXiv.2209.03464.

[10] W. Drenckhan, S. Hutzler, “Structure and energy of liquid foams” *Advances in Colloid and Interface Science* **2015**, *224*, 1–16.

[11] R. Höhler, Y. Yip Cheung Sang, E. Lorenceau, S. Cohen-Addad, “Osmotic Pressure and Structures of Monodisperse Ordered Foam” *Langmuir* **2008**, *24*, 418–425.

[12] E. Forel, E. Rio, M. Schneider, S. Beguin, D. Weaire, S. Hutzler, W. Drenckhan, “The surface tells it all: relationship between volume and surface fraction of liquid dispersions” *Soft Matter* **2016**, *12*, 8025–8029.

[13] R. Höhler, J. Seknagi, A. Kraynik, “Capillary pressure, osmotic pressure and bubble contact areas in foams” *Soft Matter* **2021**, *17*, 6995–7003.
